# Antivirals and Vaccines

**DOI:** 10.3390/ijms241210315

**Published:** 2023-06-19

**Authors:** Nuno Taveira

**Affiliations:** 1 Research Institute for Medicines (iMed.ULisboa), Faculdade de Farmácia, Universidade de Lisboa, 1649-003 Lisboa, Portugal; ntaveira@ff.ulisboa.pt; 2 Centro de Investigação Interdisciplinar Egas Moniz (CiiEM), Egas Moniz School of Health and Science, Campus Universitário, Quinta da Granja, Monte de Caparica, 2829-511 Caparica, Portugal

New antivirals are urgently needed to treat respiratory diseases caused by RNA viruses. Unlike virus-specific antivirals, host-directed antivirals—compounds that enable cells to fight viral infections—often have the advantage of being effective against multiple viruses, including drug-resistant strains (broad-spectrum antivirals) [1,2]. In addition, resistance selection is made more difficult because the compounds do not act directly on the virus, and their mechanisms of action are diverse. Unlike vaccines, these antivirals should be effective against evolving RNA virus variants, making them an important first step in the fight against new infections [2,3]. Although conceptually attractive, broad-spectrum host-directed antivirals are not yet used in the clinic, mainly due to their potential for high cellular and organismic toxicity, which can be difficult to characterise in preclinical studies; moreover, in most cases, they possess an undetermined mechanism of action (MoA). Setz et al. [4] show that pamapimod, a selective inhibitor of p38 mitogen-activated protein kinase alpha (p38 MAPKα), and pioglitazone, a broad-spectrum anti-inflammatory used to treat type 2 diabetes, strongly inhibit replication of the major SARS-CoV-2 VoCs in vitro. The activation of p38 in the lungs and heart leads to the excessive inflammation that characterises COVID-19 [5]. In addition, Setz et al. [4] show that pamapimod and pioglitazone exhibit synergistic activity when used in combination, suggesting that their combined use may represent a potential therapy for COVID-19. The effect of oral pamapimod with pioglitazone on COVID-19 development and recovery in non-hospitalised patients infected with SARS-CoV-2 is currently being investigated in a Phase II clinical trial (KIN-FAST trial, NCT05659459). On the other hand, Thaler et al. [6] show that R-propranolol exhibits broad-spectrum anti-coronavirus activity, inhibiting the replication of MERS-CoV, SARS-CoV and SARS-CoV-2 in several cell lines and in human primary bronchial epithelial cells. Using time-of-addition assays, the authors show that R-propranolol inhibits an unidentified post-entry step of the SARS-CoV-2 replication cycle, likely via a host cell factor. Propranolol, a mixture of the enantiomers R-propanolol and S-propanolol, is a non-selective beta-adrenergic antagonist (beta-blocker) used to inhibit the expression of the pro-angiogenic factor angiopoietin-like 4 (ANGPTL4) in the treatment of infantile haemangiomas [7]. R-propanolol reduces the expression of ANGPTL4, but lacks S-propanolol’s β-blocker activity. SARS-CoV-2 infection leads to pathogenic blood vessel formation in the lungs (intussusceptive angiogenesis), which is associated with the upregulation of pro-angiogenic factors such as ANGPTL4 and VEGFA [8]. Thaler et al. [6] also investigated the effect of propranolol on ANGPTL4 expression. They found that SARS-CoV-2 upregulated ANGPTL4 in endothelial and other cells, which was suppressed by R-propranolol in a process that was partially independent of the antiviral effect. R-propranolol is therefore the first example of a host-centred antiviral compound, and also has the potential to prevent detrimental host responses to viral infection.

Coronavirus infection can lead to significant mortality rates and economic losses in pets and farm animals [9]. Human infection with new and emerging zoonotic coronaviruses is a concern, so the identification of antivirals that may be effective against animal coronaviruses is a priority. Swine acute diarrhoea syndrome coronavirus (SADS-CoV) is an alpha coronavirus that causes high mortality in piglets and has a significant economic impact. SADS-CoV replicates in primary human cells, and therefore has inherent potential to spread between animal and human hosts [10,11]. There are currently no approved drugs or vaccines for SADS-CoV infection. Chen and colleagues [12] conducted a screening of more than 3500 compounds for anti-SADS-CoV activity. Gemcitabine, mycophenolic acid and its prodrug mycophenolate mofetil, methylene blue and cepharanthine were found to inhibit SADS-CoV in a dose-dependent manner. Cepharanthine and methylene blue prevented viral entry, and gemcitabine, mycophenolate mofetil, mycophenolic acid and methylene blue inhibited viral replication after SADS-CoV entry. Of note, gemcitabine [13], mycophenolic acid [14], methylene blue [15] and cepharanthine [16] have also exhibited potent in vitro activity against SARS-CoV-2 and other viruses. The broad-spectrum activity of these compounds and their established human clinical profile against COVID-19 and other human diseases [16,17,18] suggest that they may be useful in the treatment and prevention of potential outbreaks of SADS-CoV infection in pigs and humans.

Human breast milk contains secretory IgA (sIgA), macrophages, leukocytes and other components, such as lactoferrin, lysozyme, lactadherin, linoleic acid, mucins and human milk oligosaccharides (HMOs), which contribute to the overall broad antiviral properties of human milk [19]. There are over a hundred different HMOs, all derived from lactose, which can be elongated, fucosylated or sialylated. HMOs can interfere with the viral life cycle in a number of ways. For example, HMOs can act as soluble decoy receptors for rotaviruses, preventing them from binding to cell receptors and entering cells [20,21], or they can bind to the HIV-1 envelope gp120 glycoprotein and prevent the virus from binding to the dendritic cell-specific ICAM3-grabbing non-integrin (DC-SIGN) [22]. Lou et al. [23] investigated the effects of sixteen components of human milk against coxsackievirus class A type 9 isolate (CVA9). CVA9 is a non-enveloped virus with a positive-sense single-stranded RNA genome, and belongs to the family Picornaviridae, genus Enterovirus and species Enterovirus B (HEV-B). HEV-B infections usually cause mild illnesses such as hand, foot and mouth disease, but have the potential to cause more serious, life-threatening infections such as viral encephalitis and aseptic meningitis [24]. There are currently no approved drugs for infections caused by these highly variable viruses. Lou et al. [23] showed that 2’-Fucosyllactose (2’-FL) from human milk inhibited CVA9 replication in several cell lines. This effect occurred at several stages of the CVA9 life cycle, suggesting that 2’-FL acts at the cellular rather than the viral level. Enteroviruses begin their life cycle by binding to one or more cell surface receptors, leading to receptor-mediated endocytosis [25]. Receptor binding and/or pH changes in the endosomal system induce virus uncoating and the release of the viral genome from the capsid into the cytoplasm. Using time-of-addition and in silico molecular docking experiments, Lou et al. [23] found that 2’-FL binds to the attachment receptor αvβ 6 (ITGB6) and the uncoating receptors FCGRT and β2M, and exerts its inhibitory effect on CV-A9 at the level of attachment and internalisation.

Currently, it is estimated that approximately one–two million people worldwide are living with HIV-2, accounting for 3–5% of the global HIV burden [26,27]. Compared to HIV-1 infection, HIV-2 infection results in a much longer asymptomatic period, slower clinical progression and lower perinatal and sexual transmission rates. Despite the prolonged course of HIV-2 infection, early treatment initiation is recommended for all individuals, because without effective antiretroviral therapy (ART), a substantial proportion of people living with HIV-2 will develop AIDS and die [28,29]. However, many antiretroviral drugs do not work well or at all against HIV-2, which is intrinsically resistant to non-nucleoside reverse transcriptase inhibitors (NNRTIs), the fusion inhibitor enfuvirtide (T-20), some protease inhibitors (PIs) and fostemsavir, an attachment inhibitor [30,31,32,33]. In addition, to date, there have been no randomised clinical trials to guide the treatment of HIV-2 infection, particularly with regard to the choice of first- and second-line regimens. This has led to the initiation of treatment with ART regimens that are ineffective against HIV-2 [34,35]. An additional problem is that resistance to most drug classes emerges more rapidly in HIV-2 than in HIV-1 [36,37]. More effective ARVs and better use of those currently available are urgently needed for HIV-2. Moranguinho and colleagues reviewed the antiretroviral drugs presently available to treat HIV-2 infection, as well as promising drugs that are currently in development [38]. They also identified drug resistance mutations and resistance pathways that develop in HIV-2-infected individuals during treatment.

Japanese encephalitis virus (JEV), a zoonotic, mosquito-borne virus, belongs to the genus Flavivirus in the family Flaviviridae, and is closely related to West Nile virus and dengue fever virus [39]. JEV is the leading cause of vaccine-preventable encephalitis globally. Based on the phylogenetic analysis of genomic sequences, genotypes I-V (GI-V) have been described for JEV [39]. Genotype I (GI) JEV is the dominant genotype worldwide and poses a significant threat to public health security [40]. JEV causes an estimated annual burden of 68,000 cases and 20,000 deaths, and JEV encephalitis can have permanent neurological or psychological effects. There is an urgent need for highly effective vaccines and antivirals for GI JEV strains. High-throughput strategies enable the rapid screening of library compounds (drugs and antibodies) for antiviral activity [41]. Li et al. [42] developed a recombinant GI JEV expressing a Gaussia luciferase (Gluc) gene for antiviral drug screening and neutralising antibody detection. Gluc is a small molecule with a high bioluminescent signal and is naturally secreted by mammalian cells into the culture medium, enabling quantification in culture supernatant [43]. The activity levels of the antivirals nitroxoline, ARDP0006 and ribavirin against GI JEV were similar when tested using a high-throughput assay based on the recombinant construct expressing Gluc, and standard antiviral assays using the GI JEV virus. Current commercial JEV vaccines are derived from the GIII strains. Using a novel Gluc readout-based serum neutralisation assay, Li et al. [42] showed that neutralising antibodies from GIII JEV vaccines exhibit low neutralising activity against GI JEV strains, likely explaining the low protection offered by this vaccine against infection with GI JEV strains. Their results suggest that the new GI JEV Gluc reporter virus is a valuable tool for the high-throughput identification of new JEV drug candidates and for neutralisation assays against GI JEV strains.

Mayaro virus (MAYV) is an enveloped positive-strand RNA alphavirus of the Togaviridae family that causes Mayaro fever, characterised by flu-like symptoms including fever, myalgia, arthralgia and rash [44]. In rare cases, Mayaro fever causes neurological complications, haemorrhagic manifestations and death. MAYV causes sporadic outbreaks in Central and South America, but has the potential to spread globally via Aedes mosquitoes [45]. MAYV is considered a human threat because most of the world’s population is immunologically naive to it, and no specific therapeutics or vaccines have been approved for MAYV infection [45,46]. Kim et al. [47] developed viral-vectored MAYV vaccines against MAYV infection. They constructed a replication-deficient simian adenovirus (ChAdOx2) and a modified Ankara virus (MVA)-based vaccine expressing the MAYV structural polyproteins (capsid, E3, E2, 6K, and E1). Balb/c mice primed with ChAdOx2 expressing the transgenes, and boosted with the recombinant MVA, produced antibodies that bind to the E2 envelope glycoproteins of MAYV and a related alphavirus, Chikungunya virus. In addition, mice produced neutralising antibodies against both viruses, supporting the further development of these virus-vectored vaccines as a multi-alphavirus vaccine.
